# Vocal and Electric Fish: Revisiting a Comparison of Two Teleost Models in the Neuroethology of Social Behavior

**DOI:** 10.3389/fncir.2021.713105

**Published:** 2021-08-19

**Authors:** Kent D. Dunlap, Haley M. Koukos, Boris P. Chagnaud, Harold H. Zakon, Andrew H. Bass

**Affiliations:** ^1^Department of Biology, Trinity College, Hartford, CT, United States; ^2^Institute of Biology, Karl-Franzens-University Graz, Graz, Austria; ^3^Department of Neuroscience, University of Texas at Austin, Austin, TX, United States; ^4^Department of Integrative Biology, University of Texas at Austin, Austin, TX, United States; ^5^Department of Neurobiology and Behavior, Cornell University, Ithaca, NY, United States

**Keywords:** electric fish, vocal fish, mochokid catfish, social behavior, neuromodulators, hormones, communication, neural circuit

## Abstract

The communication behaviors of vocal fish and electric fish are among the vertebrate social behaviors best understood at the level of neural circuits. Both forms of signaling rely on midbrain inputs to hindbrain pattern generators that activate peripheral effectors (sonic muscles and electrocytes) to produce pulsatile signals that are modulated by frequency/repetition rate, amplitude and call duration. To generate signals that vary by sex, male phenotype, and social context, these circuits are responsive to a wide range of hormones and neuromodulators acting on different timescales at multiple loci. Bass and Zakon (2005) reviewed the behavioral neuroendocrinology of these two teleost groups, comparing how the regulation of their communication systems have both converged and diverged during their parallel evolution. Here, we revisit this comparison and review the complementary developments over the past 16 years. We (a) summarize recent work that expands our knowledge of the neural circuits underlying these two communication systems, (b) review parallel studies on the action of neuromodulators (e.g., serotonin, AVT, melatonin), brain steroidogenesis (*via* aromatase), and social stimuli on the output of these circuits, (c) highlight recent transcriptomic studies that illustrate how contemporary molecular methods have elucidated the genetic regulation of social behavior in these fish, and (d) describe recent studies of mochokid catfish, which use both vocal and electric communication, and that use both vocal and electric communication and consider how these two systems are spliced together in the same species. Finally, we offer avenues for future research to further probe how similarities and differences between these two communication systems emerge over ontogeny and evolution.

## Introduction

The neuroendocrine mechanisms underlying social behavior are daunting in their complexity. They involve many interconnected brain regions whose activities are regulated through dozens of neuroactive chemical signals acting over timescales ranging from milliseconds to years. Faced with this complexity, researchers have sought simple systems that have relatively few components whose interactions can more easily be quantified, and that can serve as models to guide studies in more complex systems. Among vertebrates, two of the most successful models have been the neural circuits underlying social communication in vocal and weakly electric fish.

In 2005, two of us (Bass and Zakon, 2005) reviewed the behavioral neuroendocrinology of distantly related teleost groups (see Nelson et al., 2016) that produce either vocalizations or electric organ discharges (EODs) and compared how their communication systems have both converged and diverged during their parallel evolution. Put briefly, both vocal and electric communication rely on hindbrain pattern generators that are relatively simple and that drive, in a one-to-one fashion, activation of peripheral effectors organs (the vocal muscles surrounding the swim bladder or the muscle-derived cells of the electric organ called electrocytes) to generate pulse-like signals. The frequency and timing of these sounds or EODs vary by sex and male phenotype (e.g., type I and II male morphs of sonic midshipman fish), and such variations are regulated largely by hormones acting as modulators in a coordinated but independent manner at multiple loci in the motor circuit.

Here, we revisit this comparison and review what has been learned in the intervening 16 years. We only briefly summarize the basics of each system since many comprehensive reviews have been published (Dunlap et al., 2017; Feng and Bass, 2017; Bass et al., 2019; Metzen, 2019). Instead, we focus on several key neural and endocrine processes that have been researched recently in both teleost systems and make direct comparisons to highlight how these analogous communication systems have evolved similar and different mechanisms. First, we summarize recent work that expands our knowledge of the neuroanatomy of circuits underlying these two communication systems. Second, we highlight several parallel studies of hormone and neuromodulator actions on these circuits. Third, we review transcriptomic studies that illustrate how contemporary molecular methods have elucidated the genetic regulation of social behavior in these two groups of fish. Finally, we describe recent studies of mochokid catfish that produce both vocal and electric signals and consider how these two systems can be spliced together and regulated in the same species.

## Brief Overview of Vocal and Electric Signaling in Fish

The use of sound and EODs as social signals has evolved in distantly related teleost groups. For details of the phylogenetic relationships of groups described in this review, we refer the reader to Nelson et al. (2016).

### Vocal Fish

**FIGURE 1:**
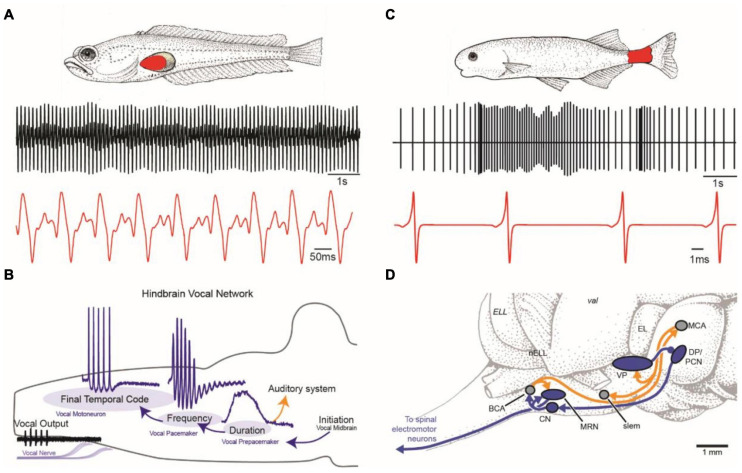
Vocal and weakly electric communication signals and pattern generating neural circuitry. **(A)** Plainfin midshipman (*Porichthys notatus*) generate sound by contracting paired muscles attached to walls of the swim bladder (red, top). Male advertisement hum with characteristic amplitude modulation shown on two timescales (bottom). **(B)** Schematic in sagittal plane showing hindbrain vocal pattern generator (blue) and corollary discharge (orange) pathways of midshipman and other toadfishes that includes three topographically separate nuclei, each coding for a different vocal attribute (adapted from Chagnaud et al., 2011). **(C)** Weakly electric mormyrids use an electric organ located in the caudal peduncle (red, top) to produce a pulsatile electric organ discharge (EOD) shown on two timescales (bottom). **(D)** Schematic in sagittal plane showing EOD pattern generating circuitry (blue) and corollary discharge pathways (orange) of mormyrid fish. BCA, bulbar command-associated nucleus; CN, command nucleus; DP, dorsal posterior thalamic nucleus; EL, exterolateral nucleus; ELL, electrosensory lateral line lobe; MRN, medullary relay nucleus; MV, medioventral nucleus; MCA, mesencephalic command-associated nucleus; OB, olfactory bulb; PCN, precommand nucleus; slem, sublemniscal nucleus; tel, telencephalon; val, valvula of the cerebellum; VP, ventroposterior nucleus. (Panels **C** and **D** adapted from Baker et al., 2013 with permission from the Journal of Experimental Biology).

Vocalization is widespread in teleost fishes (Rice et al., 2020), including some species of African electric fish (mormyroids). Our understanding of the neural mechanism underlying fish vocalization comes largely from a single group that includes toadfish and midshipman (Nelson et al., 2016). Toadfishes (order Batrachoididiformes) include close to 80 species of vocal fish found in temperate, subtropical and tropical seas that build nests in shallow waters to reproduce (Greenfield et al., 2008). Males produce their vocal signals mostly at night to attract mates and guard nests (only males provide parental care). Their vocalizations are generated by the rapid contractions (∼100 Hz at ∼16°C) of muscles attached to the walls of the swim bladder (Figure 1A).

Use of the term vocal to describe some groups of sound-producing fish was first adopted for toadfishes based on developmental and functional characters that they share, in particular, with birds, including: an effector organ dedicated to sound production, sound-producing muscles innervated by occipital (hypoglossal) nerve roots originating from motoneurons in the same caudal hindbrain location, premotor-motor circuitry with developmental origins in the same hindbrain compartments (rhombomeres), and a vocal midbrain center that gates descending input from the preoptic area-anterior hypothalamus to hindbrain pattern-generating circuitry (Bass et al., 1994; Bass, 2014). Vocal fish share some of these characters with non-avian tetrapods as well (see Bass, 2014).

Most neuroethological research on vocal fish has taken advantage of two prominent features of the highly vocal plainfin midshipman, *Porichthys notatu*s: seasonal changes in vocal behavior and alternative reproductive tactics (ARTs). Plainfin midshipman have two adult male reproductive morphs, type I and type II (Brantley and Bass, 1994). The hormonal and behavioral characters of the two male morphs diverge, while type II males and females converge. Type I males guard nests in the intertidal zone and acoustically court females at night with a multi-harmonic advertisement call known as a “hum” that lasts up to 2 h in duration and repeats throughout an evening of courtship. Type II males are smaller and neither guard nests nor produce advertisement calls. Instead, they sit near nest openings or within type I male nests where they satellite or sneak-spawn attempting to fertilize eggs. Also, like females, type II males only produce agonistic grunts (Brantley and Bass, 1994). Other non-behavioral characters (e.g., vocal muscle and motoneuron size) are also uncoupled from gonadal sex (reviewed in Bass, 1996; Feng and Bass, 2016) and may be selected upon as dissociable units. This allows for labile patterning of somatic, neural and hormonal characters over evolutionary time and gives rise to divergent intrasexual phenotypes (Goodson and Bass, 2000a; also see Bass, 1996; Lee and Bass, 2006). Although early studies showed that type I and II males follow distinct developmental trajectories (see Bass, 1996) and have non-overlapping mating tactics, later field studies revealed that small, presumably younger (see Bass, 1996), type I males act like type II cuckolders when they do not assume nest ownership, i.e., they sneak or satellite spawn (Lee and Bass, 2004, 2006).

### Electric Fish

Weakly electric fish are tropical and subtropical freshwater fish, with independent evolutionary lineages in South America (order Gymnotiformes) and Africa (order Mormyriformes) (Bullock and Heiligenberg, 1986). Together these groups contain about 500 species across both continents. Most species produce weak electric discharges from modified muscle cells, electrocytes, located in the electric organ of the tail, and they detect these discharges through specialized electroreceptors located across the body (Figure 1C). They emit their EOD continuously, and in many species, they enhance their EOD at night, when they are most active.

Weakly electric fish use their EOD for sensing objects around them (electrolocation), but more relevant to this review, it is their primary modality of social communication (electrocommunication). The EOD conveys information about the sex and motivational state of an individual. For example, in most species, males and females differ in the frequency or wave form of their continuous EOD, and, during aggression and courtship, they produce brief frequency and/or amplitude modulations of the EOD (e.g., chirps and rises) that last milliseconds to seconds (reviewed in Dunlap et al., 2017).

### Comparison

One major advantage of studying both vocal and electric fish is that their communication behaviors can be readily characterized by a finite set of easily quantified physical attributes. In both modalities, the signals vary in frequency (repetition rate), duration, and frequency/amplitude modulations, and these signal parameters commonly differ by sex and vary according to social context (Caputi et al., 2005; Bass et al., 2015). However, communication signals differ between vocal and electric fish in at least three ways. First, vocal fish intermittently produce their signals with important variation in the call duration while electric fish continuously generate their signals. Second, vocal fish produce their acoustic signals only for communication while electric fish use their EOD for the dual functions of electrocommunication and electrolocation. Finally, electric fish can generate salient variation in the waveform of their signal while the vocal signals vary little in waveform.

In general, the frequency of the signal in both groups is established by a hindbrain pattern generator. Modulations of this baseline rhythm, such as variations in frequency or call duration, arise from midbrain inputs to the pattern generator. As pointed out previously (Bass and Zakon, 2005), the two systems differ in the role of the effector organ (vocal muscle or electric organ) in shaping the signal. In vocal fish, the vocal muscles do not modulate the waveform, but in electric fish, the electrocytes of the electric organ play a crucial role determining the shape of the signal (e.g., the number of phases and the duration of each phase). Below, we further compare the neural circuits underlying these two communication systems.

## Neural Circuits Underlying Vocal and Electric Signaling

Comparisons of the neural circuitry in vocal and electric fish date back to the pioneering work of M.V.L. Bennett and colleagues in the 1960s and 1970s (Bennett, 1971a,b), who documented the electrotonic coupling between motoneurons in both systems. Over time, researchers revealed further similarities in structure-function organization shared by the pattern generating circuits underlying vocal and electric signal production (Bass, 1986, 1989; Bass and Baker, 1997; Bass and Zakon, 2005). This included the salient role of temporal precision in vocal muscle and electric organ activation, the location of pattern generating circuitry in the hindbrain near the spinal cord boundary, and the co-evolution of vocal motor and electromotor systems with their respective sensory systems to enhance sensory-motor coupling. Here, we further compare the pattern generating circuits between vocal and electric teleosts given the most recent studies of neural mechanisms for generating and perceiving communication signals.

### Neural Circuitry Generating Vocal and Electric Signals

In signal generation, both vocal and electric modalities require precision in the temporal and spatial domains and sufficient energy for conspecific communication (Figures 1A,C). Because sound degrades in amplitude in the aquatic medium, especially in shallow water, most vocal fish face conditions unfavorable for long distance communication. Similar constraints exist for electric signals, which attenuate spatially to an even greater degree (Brenowitz, 1986). The solution in both modalities for extending their communication range is to generate high amplitude signals by synchronizing the oscillations of cells in the effector organs (muscle fibers of the vocal muscles and electrocytes of the electric organ) (Bennett et al., 1967b; Bass and Baker, 1990). Such synchrony is achieved in both systems by several specializations, including reduction in the number of motoneurons innervating the effector organ or by coupling motoneuron activation *via* presynaptic inputs and/or gap junctions (Bennett et al., 1967b; Bennett, 1971a; Bass and Baker, 1990; reviewed in Caputi, 2020). An extreme example of reduced central control is in electric catfish (*Malapterurus)*, which have a single bilateral pair of motoneurons, each one innervating several millions of ipsilateral electrocytes (Bennett et al., 1967a; Bennett, 1971a).

The production of both vocal and electric signals relies on activating neurons at high frequencies (∼50–1100 Hz). Thus, a potential problem in both systems is erratic, spontaneous firing, which would disrupt synchrony. As one adaptation to prevent such unregulated activity, motoneurons in both systems have low input resistance, and thus require coherent synaptic input to fire action potentials. In this way, the motoneurons may be considered “followers.” Recent studies of vocal fish (Chagnaud et al., 2021) and other vertebrate vocal (Lawton et al., 2017) and locomotor systems (Song et al., 2016; Matsunaga et al., 2017), however, suggest that the influence of motoneurons on premotoneurons *via* gap junctions gives them greater importance in patterning the activity of the effector organ than merely following premotor input (reviewed in Barkan and Zornik, 2019). Furthermore, motoneurons in vocal and electric systems are adapted to phasic input, preferentially firing at the onset of intracellular current influx. This makes them ideally suited to respond to short pulses of current flux and repetitive activity (Chagnaud et al., 2012). This adaptation clearly facilitates high frequency oscillatory-like firing, another common feature of motoneurons in both vocal and electric modalities.

If motoneurons are followers, who do they follow? In vocal fish and gymnotiform electric fish, neurons with pacemaking capabilities project directly (vocal) or indirectly (electric) to the motoneurons. In vocal fish, pacemaker neurons show intrinsic properties enabling voltage-dependent oscillatory behavior, but the pacemaker neurons themselves do not generate rhythms in the absence of synaptic input (Chagnaud et al., 2011). By contrast, in electric fish, relay neurons receive input from pacemaker neurons and “relay” patterning information to the motoneurons (Grant et al., 1986; Carlson, 2002). In one group of electric fish, the apteronotids, electromotor neurons have intrinsic rhythmic firing independent of sensory or midbrain input; their axons form the electric organ itself and are the source of the EOD as they lack the muscle-derived electric organ found in other electric fish (Dye and Heiligenberg, 1987; Shifman et al., 2020). This marked difference between vocal and electric fish in pacemaker circuitry correlates directly with how they control signal duration. While vocal fish modify the duration of different call types, electric fish instead mainly modulate the brief pauses between discharges. The duration of pauses can last from milliseconds to seconds, but because the EOD is used for electrolocation as well as electrocommunication, this system is never fully “turned off.”

Several studies describe a variety of inputs to the vocal (Forlano et al., 2014; Rosner et al., 2018; Timothy and Forlano, 2020) and electromotor (Borde et al., 2020) pattern generating circuits in the hindbrain (Figures 1C,D). One interesting aspect of a recent study investigating the neurophysiological correlates of such inputs is the identification of gap junction coupled, glycinergic neurons within the vocal circuit (Chagnaud et al., 2021). These neurons are interesting in light of an early study (Pappas and Bennett, 1966) that provided evidence for inhibitory action onto vocal motoneurons. A neurophysiological study (Chagnaud et al., 2021) revealed the importance of glycinergic input to motoneurons in synchronizing the vocal motor output. In addition, this study showed that gap junctional coupling is essential to activate these glycinergic neurons and that vocal premotor neurons are not only excited by gap junctional coupling (Pappas and Bennett, 1966; Bass and Baker, 1990), but that gap junctional coupling is indeed sufficient to activate premotoneurons (Chagnaud et al., 2021). Such coupled glycinergic neurons could also contribute to the temporal patterning of EODs in the hindbrain pattern generator, but this remains to be investigated.

### Neural Circuitry for Reception of Vocal and Electric Signals

In toadfish, vocal communication depends on the detection of sound waves by inner ear otolith organs, especially the saccule (Fay and Popper, 2012). The hearing range of fishes is generally limited to <1 kHz, except in species with accessory organs (e.g., Weberian ossicles) that permit a higher frequency detection (Braun and Grande, 2008). In vocal fish, auditory neurons, especially those in the hind- and midbrain, encode vocalization attributes such as frequency content (e.g., encoded as best frequency), patterns of amplitude and frequency modulation, and the onset and overall duration of sound waves (Bass and Lu, 2007; Fay and Edds-Walton, 2008). Sound-producing and sound-perceiving circuits are not fully separated, as vocal-auditory coupling at different levels of the auditory system ensures that the latter is informed about one or more acoustic characters (Weeg et al., 2005; Chagnaud and Bass, 2013). For example, the vocal pattern generator in midshipman fish (Figure 1B) relays information about vocal duration from a prepacemaker nucleus to a separate hindbrain population that directly innervates the auditory epithelium of the inner ear (Chagnaud and Bass, 2013).

In electric fish, similar information is coded at peripheral and central levels. Electrosensory neurons have response properties similar to auditory neurons of vocal fish. The electroreceptors are tightly tuned to the dominant frequency in the fish’s EOD. In species where the males and females differ in EOD frequency, the tuning of electroreceptors show corresponding sexual differences. As with the vocal system, information about ongoing EOD activity is transmitted to sensory structures from the motor command system (Figure 1D) *via* either peripheral reafference (gymnotiformes, *Gymnarchus*) or central corollary discharge (mormyrids) pathways (Perks and Sawtell, 2019; Fukutomi and Carlson, 2020). Extensive literature dating back to the early 1960s (Bennett, 1971b; Heiligenberg, 1977) documents this electrosensory processing, often with comparisons to audition, and was recently reviewed elsewhere (Carlson, 2004; Caputi, 2017; Carlson et al., 2019).

Despite coding for different behaviors, the vocal and electric pattern generators thus share several fundamental features such as oscillatory activity, synchrony and neural precision on the motor patterning side, as well as feature extraction of sensory stimuli and a strong connection between the motor and the sensory circuits. Future studies are needed to evaluate whether those shared general attributes are reflected in individual neurons by employing similar ion channels in the neurons coding for these two modalities.

## Neuromodulatory and Hormonal Regulation of Communication and Social Behavior

The previous review comparing these two teleost models (Bass and Zakon, 2005) emphasized how steroid hormones act on the underlying neural circuits described above to achieve long-term changes (days to months) in communication behavior associated with season, sex and male phenotype. Since then, much work has focused on how other hormones and modulators work in combination (or in parallel) with steroids to regulate social behavior on more rapid time scales, ranging from minutes to days. Below, we summarize studies on how serotonin, AVT, melatonin and melanocortins influence communication and social behavior and how steroids can regulate these circuits through newly described mechanisms. These recent studies reinforce previous work showing how hormones coordinate the responses to predictable changes in the physical environment (e.g., behavioral responses to diel and annual cycles). In addition, these recent studies underscore the role of hormones and neuromodulators in coordinating the response to unpredictable and dynamic social environments.

### Serotonin

Most communication signals are specific to social context. In the vocal and electric modalities, this specificity is achieved by modifying particular signal attributes (e.g., duration, spectral content, amplitude or frequency modulation). Neuromodulators acting at select loci in neural circuits contribute to such plasticity in signal production. In frog (Rhodes et al., 2007; Yu and Yamaguchi, 2010; Kelley et al., 2020) and bird vocal systems (Wood et al., 2011), serotonin (5HT) is one such modulator. The organization of the serotonergic system is highly conserved among teleost fishes (Lillesaar et al., 2007; Lillesaar, 2011; Lillesaar and Gaspar, 2019), and the widespread distribution of serotoninergic neurons in brains of this more ancestral vertebrate group suggests that 5HT may have played an important role in the evolution of neuroendocrine mechanisms regulating vertebrate communication behavior.

#### Vocal Fish

Although no one has yet investigated the behavioral or physiological effects of 5HT on vocal production in teleosts, several studies, primarily in toadfish, have described the distribution of 5HT and the projections of serotonergic neurons in vocal control regions of the brain, from higher order centers in the fore- and midbrain down to the pattern generator in the hindbrain (Rosner et al., 2020; Timothy and Forlano, 2020). Serotonergic projections to vocal-associated neurons are well identified, especially in neurons connected to the vocal pattern generator. However, we have only indirect evidence of such projections to vocal-associated neurons in higher brain areas. Such evidence could be demonstrated by combining 5HT immunocytochemistry with immediate early gene expression during vocal activity, as described previously for catecholaminergic neurons (Petersen et al., 2013).

In their recent study of 5HT distribution in the midshipman brain, Timothy and Forlano (2020) found that all currently known regions within the forebrain vocal-acoustic complex and the midbrain acoustic complex (each containing several brain nuclei) are characterized by serotonergic presence. By taking advantage of extensive transneuronal transport, investigators have mapped the hindbrain vocal pattern generator in toadfishes. Application of either neurobiotin or biocytin to a single vocal nerve leads to labeling of vocal motoneurons as well as premotor populations of pacemaker and prepacemaker neurons (Bass et al., 1994; Chagnaud and Bass, 2014). This feature likely depends on extensive gap junction coupling between neurons (see Bass et al., 1994; Chagnaud et al., 2011, 2012). Both 5HT immunoreactive somata and fibers are present in the vocal motor nucleus (VMN) (Rosner et al., 2018, 2020; Timothy and Forlano, 2020), which consists mainly of motoneurons innervating the vocal muscles (Bass, 1985). Transneuronally coupled neurons were not 5HT-positive (and thus likely not coupled *via* gap junctions). Since the VMN synchronizes motoneuronal firing and thereby plays a large role in determining call amplitude (Chagnaud et al., 2012), 5HT in the VMN may contribute to the modulation of call amplitude, which is a distinctive feature of toadfish vocalizations.

The neurons of the vocal pacemaker nucleus (VPN) that code for the fundamental frequency of toadfish vocalizations (Chagnaud et al., 2011) are also characterized by 5HT projections to the somata and to the VPN dendritic tree. Since frequency modulation is especially prominent in some vocalizations, 5HT could also act on call pulse repetition rate/fundamental frequency. The third main component of the toadfish vocal pattern generator, the vocal pre-pacemaker (VPP), also receives 5HT-ir projections, and 5HT ir-positive neurons are located in its immediate vicinity. 5HT may modulate call duration, which is regulated by the VPP (Chagnaud et al., 2011). Due to the major differences in call durations between vocalizations, which range from a few milliseconds to hours, neuromodulators could participate in generating such call diversity.

The well-mapped distribution of serotonergic neurons in the vocal motor system strongly suggests that 5HT acts at many loci to independently modulate different features of the acoustic call. However, it will be important to follow these neuroanatomical studies with corresponding physiological studies that demonstrate the full effects of 5HT. The description of 5HT distribution in vocal associated areas bears the caveat that neurons in some of these brain areas (e.g., POA or periaqueductal gray) are also known to be associated with other behaviors.

#### Electric Fish

In contrast to the thorough neuroanatomical description of the serotonergic system in vocal fish, the distribution of 5HT has not been investigated as deeply in electric fish. However, many physiological studies in gymnotiform electric fish using agonists and antagonists of serotonin receptors have demonstrated that 5HT is a widespread modulator of electric signaling and social behavior, exerting its influence on both the production and reception of the EOD at many different circuit levels (Zubizarreta et al., 2012; Marquez and Chacron, 2020). Depending on the context and the species, 5HT can modify EOD pulse amplitude and shape, EOD modulations, “chirps,” as well as the electrosensory perception of the EOD. Moreover, the overall outcome of dominance interactions is heavily influenced by 5HT, and species differences in aggressive behavior correlates with evolved differences in the serotonergic system.

In *Brachyhypopomus pinnicaudatus*, males normally increase EOD duration (repolarization of the second phase) and EOD amplitude as they enter the dark phase of the light cycle (Stoddard et al., 2007). When presented with a conspecific male, these parameters increase even more so, perhaps as a way of exaggerating the range and “masculinity” of their signal. This exaggeration of the circadian oscillation is mimicked with peripheral injections of 5HT, which act within minutes *via* 5HT2 and/or 5HT1A receptors to increase EOD amplitude and duration (Stoddard et al., 2003; Allee et al., 2008). However, *in vitro* 5HT application directly to isolated electrocytes and the spinal cord has no effect (Markham and Stoddard, 2005), suggesting that 5HT acts centrally, perhaps through regulating pituitary secretion of melanocortins (ACTH and alpha-MSH), which then act directly on electrocytes (see section “Regulation of Diel Patterns of Signaling”). Both the pre-optic area (POA) and hypothalamus, whose activity influences pituitary secretion, densely express 5HT (Johnston et al., 1990) in the neuron terminals, and this may represent an endogenous pathway for serotonin regulation of the EOD via melanocortins.

At higher levels in the neural circuit, 5HT appears to exert an inhibitory action on EOD modulations during aggressive interactions. In *Apteronotus leptorhynchus*, the midbrain pre-pacemaker nucleus (PPn-C) initiates the production of “chirps” – rapid frequency/amplitude modulations of the EOD – *via* monosynaptic inputs to the pacemaker nucleus (PN). These chirps, especially the short duration type 1 chirps, are produced most vigorously during male aggression. Males injected intracerebrally with 5HT reduce their chirping (Maler and Ellis, 1987), and females, which chirp much less than males, have much greater expression of 5HT in the PPn-C (Telgkamp et al., 2007). In addition, among females, subordinate individuals have more 5HT in the PPn-C than did dominants. Pharmacological manipulations indicate that this inhibitory action of 5HT on aggressive chirps is mediated through 5HT2 receptors (Smith and Combs, 2008). Interestingly, 5HT may act through 5HT1A receptors to increase the production of type 2 chirps, which are produced by males during courtship. Together, these studies thus indicate that 5HT acts on the PPn-C to contribute to sexual differences and context-specific expression of chirps.

Several sets of studies have demonstrated that, in addition to inhibiting the production of chirps used in same-sex aggression, 5HT simultaneously enhances perception of same-sex stimuli (Deemyad et al., 2013; Marquez and Chacron, 2020). Using fast-scan cyclic voltammetry, Fotowat et al. (2016) showed that 5HT is released in the electrosensory-lateral line lobe (ELL) of the hindbrain in response to stimuli mimicking a conspecific male. Experimental elevation of local 5HT enhances the sensitivity of pyramidal neurons in the ELL and promotes burst firing of these neurons (Deemyad et al., 2013; Márquez et al., 2013). 5HT likely increases pyramidal cell excitability by binding to 5HT2 receptors (Larson et al., 2014) and downregulating the potassium channels that contribute to the spike afterhyperpolarization. The same 5HT treatments that enhance this electrosensory sensitivity simultaneously inhibit chirp production (Deemyad et al., 2013). Thus, the authors of this work proposed that, overall, 5HT serves a “shut up and listen” function that minimizes aggression during same-sex interactions and contributes to social subordination.

**FIGURE 2:**
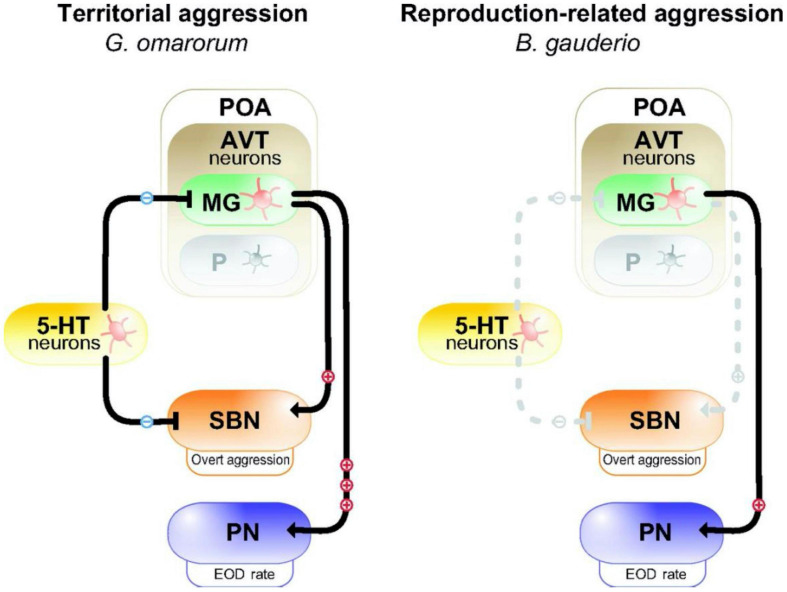
Model for the neuromodulation of aggression by AVT and 5-HT in dominants of two species of electric fish with different forms of aggression. In dominant *Gymnotus omarorum* **(left)**, which displays territorial aggression year round, AVT magno-gigantocellular neurons (MG) in the pre-optic area (POA) strongly activate (+++) the pacemaker nucleus (PN) to increase EOD rate, and weakly active (+) overt aggression through the social behavioral network (SBN). 5-HT neurons inhibit (−) overt aggression. In dominant *Brachyhypopomus gauderio* **(right)**, which shows only reproduction-related aggression during the breeding season, AVT-containing MG cells of the POA weakly (+) increase EOD rate, but have no effect (dashed line) on overt aggression. 5-HT has no effect on aggression. The parvocellular cells (P) in the POA do not participate in regulating aggression in dominants of either species. Reproduced/adapted with permission from the Journal of Experimental Biology, Silva et al. (2013).

The inhibitory action of 5HT on aggression typical of many vertebrates is exhibited in the interspecific comparison of two electric fish species (Figure 2; Zubizarreta et al., 2012; Silva et al., 2013). *Gymnotus omarum* is especially aggressive, and both males and females quickly attack intruders in the non-breeding as well as the breeding season. Associated with this high level of aggression, basal 5HT activity levels in the telencephalon of *G. omarum* are relatively low, and these levels fall even further in both combatants following staged encounters (Zubizarreta et al., 2012). Aggression is inhibited by the 5HT agonist 8-OH-DPAT, indicating that 5HT likely acts through 5HT1A receptors. By contrast, *Brachyhypopomus gauderio*, which is overall less aggressive and exhibits aggression only by males in the breeding season, has relatively high telencephalic 5HT activity. Following territorial disputes, 5HT activity increases but only in subordinates. The anti-aggressive actions of 5HT in this species does not occur *via* 5HT1A receptors. These species differences in the regulation of aggression indicate that evolutionary changes in the serotonergic system may have contributed to species diversification in patterns of social behavior.

#### Comparison

The serotonergic systems of vocal and electric fish have been studied largely through different approaches: neuroanatomical in vocal fish and physiological/behavioral in electric fish. Nonetheless, 5HT appears to have widespread influence on the communication behavior of both groups. In vocal fish, 5HT likely acts directly on nuclei in the hindbrain pattern generator as well as indirectly through fore- and midbrain inputs. In electric fish, it acts on the midbrain prepacemaker nuclei or on the hypothalamic regulation of melanocortins to regulate the production of electrocommunication signals. In addition, 5HT modifies neural activity in the electrosensory-lateral line lobe of the hindbrain to enhance electrosensory perception. Anatomical evidence of serotonergic projections in the auditory processing nuclei in vocal fish (Timothy and Forlano, 2020) suggest that auditory perception might be similarly affected. Finally, it appears that, among electric fish, the diversity of serotonergic receptors contributes to species differences in overt aggressive behavior as well as social communication.

### Arginine Vasotocin

Across vertebrates, social behavior is greatly influenced by the nonapeptide arginine vasotocin (AVT) and its homologs, and evolution within the AVT system has likely contributed to behavioral diversification among vertebrates (Goodson, 2008, 2013). Consistent with this general trend among vertebrates, AVT has potent effects on the social behavior in vocal and electric fish, and variations within the AVT system contribute to inter- and intra-specific differences in social behavior.

#### Vocal Fish

In vocal fish, the action of AVT has been studied most in an electrophysiological preparation in which AVT can be applied to specific brain regions while monitoring “fictive” output from the vocal nerves (Goodson and Bass, 2000a). In type I males of the plainfin midshipman, AVT decreases fictive call duration when it is injected directly into the preoptic area (POA) and anterior hypothalamus while application of an antagonist of the V1a AVT receptor increases call duration. When injected into the midbrain, AVT decreases the number of calls without affecting duration. In contrast to these actions in type I males, AVT has no effect on the vocal motor output of type II males and females. Instead, another nonapeptide, isotocin (an oxytocin homolog), exerts a potent inhibitory effect on vocal output. Immunoreactive AVT and isotocin fibers are found in neurons of many fore- and midbrain regions that influence vocal production, including the POA, the periaqueductal gray and the paralemniscal midbrain tegmentum. Interestingly, there appears to be no labeling in the hindbrain regions that are most directly related to vocal production (Goodson and Bass, 2000b; Goodson et al., 2003).

Although fish of different sex and morphotype show divergent responses to experimental manipulations of these nonapeptides, they show similar nonapeptide distribution in brain. This suggests that the different responses to exogenous AVT is likely attributable to differences in the density or distribution of their receptors.

#### Electric Fish

In electric fish, AVT modifies both agonistic behavior and electric signal production, and the specific effect varies widely by sex, dominance status, and species (Bastian et al., 2001; Perrone et al., 2010). In male *Apteronotus leptorhynchus*, AVT injection inhibited the production of aggressive chirps (type 1), however, this same treatment stimulated production of male courtship chirps (type 2). AVT had no apparent effect on chirping in females (Bastian et al., 2001). Thus, in this species, the action of AVT is specific to both sex and signal type. The mechanism and site of AVT action on chirping is unknown, however, AVT has been localized in the POA (Johnston and Maler, 1992) and there are abundant known connections between the POA and the PPn-C, the brain region that controls chirping.

In the gregarious species *Brachyhypopomus gauderio*, in which aggression is naturally confined to the breeding season, AVT administration to males during the breeding season increased diurnal EOD rate, which is a signal characteristic of dominant males (Figure 2; Perrone and Silva, 2016). Double labeling for AVT and an immediate early gene, FOS, showed that many neurons in the POA that express AVT become active specifically when a male is exposed to a female (Pouso et al., 2019). AVT neurons project from the POA to the PN, where AVT binds to V1a receptors to increase firing rate of pacemaker neurons (Perrone et al., 2014; Pouso et al., 2017). These studies demonstrated a positive effect of AVT on male sexual signaling. Interestingly, in this species, AVT had little effect on overt aggression (i.e., fighting) (Perrone and Silva, 2016).

In the solitary species *Gymnotus omararum*, which displays aggression year-round and mostly in the context of territorial disputes, the effect of AVT is notably different than in the gregarious *Brachyhypopomus gauderio* (Figure 2). AVT administration has little effect on basal EOD rate (Silva et al., 2013; Perrone and Silva, 2018). However, it modifies the production of submissive electric signals in a status-dependent manner: AVT increases submissive signaling in subordinates while showing no effect in dominants. As an additional example of species-specific actions, AVT increases the motivation for overt aggression in *Gymnotus*, but has no effect on overt aggression in *Brachyhypopomus*. Although these two electric fish species differ markedly in their AVT regulation of electrocommunication and aggression, they show no apparent differences in the distribution of AVT in the brain (Pouso et al., 2017). Thus, just as in vocal fish, variation in the behavioral response of electric fish to AVT is likely due to the variation in the distribution of receptors.

#### Comparison

It is clear from studies on both vocal and electric fish that, while AVT is an important regulator of social behavior, its effects are highly context-dependent; its actions vary considerably in intrasexual (plainfin midshipman, *Gymnotus*), intersexual (*Apteronotus*) and interspecific (*Gymnotus* vs. *Brachyhypopomus*) comparisons. Vocal and electric fish both have AVT receptors sensitive to V1a receptor antagonists in the neural circuitry underlying social communication. However, in both teleost models, behavioral differences (intrasexual and interspecific) are not related to any corresponding differences in AVT distribution in the brain.

In addition to these similarities, there is an apparent difference as well. In vocal fish, AVT tends to inhibit production of communication signals, acting at the level of the fore- and midbrain. In electric fish, it inhibits production of some signals (Type I chirps in *Apteronotus*) but stimulates other signals (type II chirps in *Apteronotus*, EOD rate in *Brachyhypopomus*, submissive signaling in *Gymnotus*). Finally, in vocal fish, AVT neurons are not found in the vocal control nuclei of the hindbrain, but, in at least some electric fish, AVT neurons are located in the hindbrain pattern generator as well as within the fore- and midbrain.

### Regulation of Diel Patterns of Signaling

Both vocal and electric fish are socially most active at night and emit their communication signals in pronounced daily cycles. Several sets of studies in both groups have explored the role of melatonin acting in the brain or melanocortins acting in the periphery in regulating these diel cycles. In diverse vertebrate taxa, melatonin is released from the pineal gland in the dark phase of the photic cycle and serves as the main time-regulating hormone. Melanocortins [e.g., adrenocorticotropin (ACTH) and alpha-melanocyte stimulating hormone (alpha-MSH)] are secreted from the pituitary into the blood where they coordinate daily cycles in peripheral tissues.

#### Vocal Fish

**FIGURE 3:**
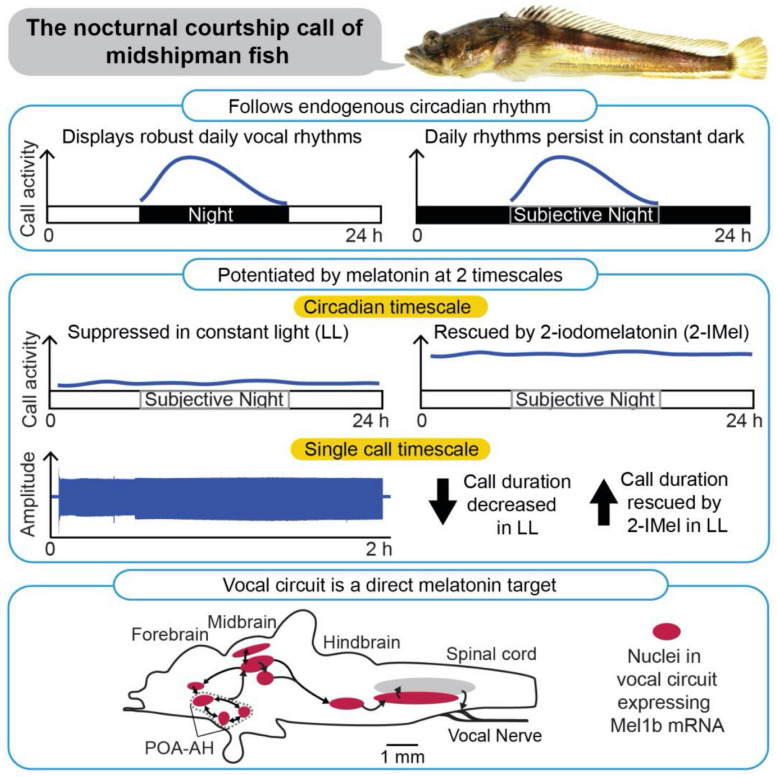
Patterning of social acoustic signaling in the nocturnally breeding, plainfin midshipman fish (*Porichthys notatus*) at multiple timescales, from day–night rhythms to call temporal properties. **(Top)** Courtship vocalizations exhibit an endogenous circadian rhythm under constant dark conditions. **(Middle)** Courtship vocalizations are suppressed under constant light (LL), but systemic delivery of melatonin analog (2-IMel) rescues their daily occurrence (circadian timescale), including their duration (single call timescale). **(Bottom)** Sagittal view of midshipman brain (rostral is to the left) depicting robust expression of melatonin receptor lb mRNA transcripts in evolutionarily conserved neuroendocrine and vocal networks, including the preoptic area-anterior hypothalamus (POA-AH). From Feng and Bass (2016).

As nocturnal fish, male plainfin midshipman broadcast advertisement calls repeatedly throughout the night. Feng and Bass (2016) demonstrated that this rhythmic display of courtship vocalization is synchronized by light conditions (Figure 3). Beginning with fish housed in a 15L: 9D light cycle that mimics the photic conditions in which the fish normally vocalize, they then transferred one group of fish to constant dark (DD) and another group to constant light (LL) conditions. DD fish displayed humming behavior during the subjective night, demonstrating an endogenous circadian rhythm. However, this cycle was disrupted in LL fish (Feng and Bass, 2016).

Subsequent studies showed that the nocturnal increase in vocalization is mediated by endogenous melatonin (Feng and Bass, 2014, 2016; Feng et al., 2019). DD fish stopped calling when the endogenous actions of melatonin were blocked pharmacologically (Feng and Bass, 2014). Conversely, LL fish resumed their cycles of humming when treated with a melatonin analog (Feng and Bass, 2016). The light-dependence of vocal behavior is paralleled by the *in vivo* excitability of the underlying neural circuits. In an intact neurophysiological preparation where fictive calls were evoked through electrical microstimulation of midbrain nodes in the vocal network, constant darkness decreased the threshold for evoking calls and increased call duration. These effects were reversed by melatonin receptor antagonists. By contrast, constant light decreased excitability and call duration, measures that were reversed by treatment with melatonin agonists (Feng and Bass, 2014).

Additional *in situ* hybridization studies demonstrated that melatonin receptor (mel1B) has widespread distribution within the brain, including in the hindbrain pattern generator circuit [i.e., the VPP, the vocal pacemaker (VPN)] and in fore- and midbrain nuclei (e.g., POA, the periaqueductal gray) that contributes to vocal production (Figure 3; Feng et al., 2019). While this distribution of receptors indicates that melatonin could act directly on the vocal circuitry, it is important to note that these receptors also colocalize with other key neurochemical regulators of vocal production (e.g., steroid hormones, aromatase, and AVT), and that melatonin likely works in combination with these molecules to regulate diel and perhaps annual cycles of calling behavior (see Feng et al., 2019).

Together, the above behavioral, neurophysiological and neuroanatomical studies support the hypothesis that the stimulatory effect of darkness on vocalization is mediated by endogenous melatonin. One curiosity is that, although melatonin stimulates vocal behavior, fish vocalize most vigorously during the summer, when the duration of night and the period of elevated melatonin levels are short. This suggests that, in these nocturnal fish, the magnitude of melatonin secretion or sensitivity of the circuits to melatonin, rather than the duration of melatonin secretion, drives seasonal changes in calling behavior. As the summer breeding season approaches, fish migrate from deep, cold water to the warmer waters of the intertidal zone (Feng and Bass, 2014). This temperature increase may enhance nocturnal melatonin secretion or potentiate its effects in the vocal motor circuitry.

#### Electric Fish

Because electric fish can navigate, locate prey and communicate using their electrosensory system alone, they can perform most of their activities in complete darkness, and virtually all species examined are highly nocturnal (Bullock and Heiligenberg, 1986). Additionally, in several species, multiple features of their EOD (e.g., frequency or amplitude) are enhanced at night. Such changes enable them to sample their environment more frequently, expand the range of their signal and, in some cases, exaggerate the “maleness” of their signal. Conversely, a daytime decrease in these parameters lowers the substantial energetic cost of electrogenesis during the period when they are less apt to use electrolocation and electrocommunication (Salazar and Stoddard, 2008; Salazar et al., 2013).

Several species that emit a pulse discharge show nocturnal increases in EOD rate (Zupanc et al., 2001; Silva et al., 2007; Stoddard et al., 2007; Migliaro and Silva, 2016). Such rhythms are maintained even in constant photic conditions in the laboratory, indicating an intrinsic circadian organization in the activity of the hindbrain pacemaker. In one species, *Gymnotus omarorum*, this circadian rhythm in the EOD persists in field conditions even after controlling for diel changes in light, temperature and locomotion. However, these field studies also indicate that social interactions help synchronize the diel changes in EOD rates (Migliaro et al., 2018).

The nocturnal increase in EOD rate is likely mediated through endogenous fluctuations in melatonin, since a melatonin receptor antagonist eliminates the rhythm (Migliaro and Silva, 2016). Because melatonin receptor distribution has not yet been mapped, it is unknown whether melatonin binds directly to neurons in the pacemaker nucleus or whether it acts indirectly at other sites or through other neurochemical mediators. However, it is unlikely that endogenous AVT fluctuations participate in circadian patterns of EOD rate, since AVT receptor antagonists have no effect on this rhythm.

In addition to these EOD rate changes originating in the hindbrain pacemaker, EOD amplitude also fluctuates in a circadian pattern, indicating that the biophysical properties of the electrocytes in the periphery also cycle daily (Stoddard et al., 2006). In *Sternopygus*, the day–night cycle in EOD amplitude recorded from intact fish is paralleled by daily fluctuations in action potential amplitude of electrocytes measured *in vitro*. Electrocytes harvested at night generate higher amplitude action potentials than those harvested from the same individual during the day (Markham et al., 2009b). This diel cycle in action potential amplitude (and thereby EOD amplitude) is accomplished at the cellular level by trafficking sodium channel proteins between the electrocyte membrane during the night and back into intracellular vesicles during the day. Changes at all these levels – whole organism EOD, electrocyte excitability and ion channel trafficking—can be accomplished within minutes by *in vivo* and *in vitro* treatment with adrenocorticotropic hormone (ACTH), which is known to fluctuate in a circadian pattern in many teleost fish. Thus, researchers have traced the hormonal regulation of circadian changes in the communication behavior of these fish to identified subcellular processes that underlie the output of the peripheral effector organs.

In *Brachyhypopomus*, EOD shape along with EOD amplitude vary in a circadian pattern (Stoddard et al., 2007). Specifically, the second phase of the EOD, which is already broader in males than in females, becomes even broader at night. Thus, males further masculinize their EOD during periods when they are most engaged in social behavior. In a manner similar to that of *Sternopygus*, melanocortins (alpha MSH and ACTH) in *Brachyhypopomus* act directly on electrocytes *via* a cAMP/PKA phosphorylation pathway that regulates the electrocyte biophysics, in this case by altering kinetics of both voltage-gated sodium and potassium channels (Markham and Stoddard, 2005, Markham et al., 2009a).

#### Comparison

These studies over the past decade have demonstrated that melatonin likely plays a crucial role in diel rhythms of social signaling in both vocal and electric fish. Both groups show circadian patterns of signaling behavior that can be modified by manipulation of melatonin levels. In vocal fish, melatonin appears to act primarily on the duration of calls while in electric fish it acts on the EOD rate. Because midshipman fish live in the temperate zone and inhabit shallow intertidal waters during the breeding season, they experience seasonal changes in daylength and thus seasonal changes in melatonin may act in combination with reproductive hormones to regulate annual cycles of signaling behavior. By contrast, electric fish generally live in tropical regions where daylength changes are less detectable. Consequently, they may be less likely to use melatonin for regulating annual cycles of signaling. In electric fish, diel changes in signaling are also regulated by melanocortins as well as melatonin, while melanocortins have not been examined in vocal fish. These hormones act peripherally to control diel patterns in EOD amplitude and shape by acting on regulation and trafficking of ion channels in the electrocyte membrane.

### Aromatase

One focus of a previous comparison between vocal and electric fish (Bass and Zakon, 2005) was the role of sex steroids produced by the gonads in long-term regulation of communication behavior during the breeding season. Since then, studies in both vocal and electric fish have demonstrated that rapid metabolism of steroids by the brain can be an important regulator of social behavior during the breeding season and, in electric fish, the non-breeding season as well. In these recent studies, the emphasis has been on the distribution and action of the steroidogenic enzyme aromatase in the brain. Aromatase converts testosterone to estradiol, and in doing so, it influences the local production and action of steroids on neural circuits controlling behavior. Compared to other vertebrates, teleost fish, including vocal and electric fish, have exceptionally high brain levels of aromatase (Forlano et al., 2006), suggesting that the brain is an important site of steroid metabolism.

#### Vocal Fish

In plainfin midshipman, aromatase is prominently expressed in brain regions controlling vocal production, including the POA, anterior and ventral tuberal nuclei in the forebrain, the periaqueductal gray in the midbrain, and the vocal pre-pacemaker nucleus (VPN) and VMN in the hindbrain (reviewed in Forlano et al., 2015). Aromatase expression varies across seasons, sexes and male phenotype, indicating a regulatory role in vocal signaling (reviewed in Shaw, 2018). Aromatase increases in the POA and VMN in females during the pre-nesting period and in type II males during the nesting period. Moreover, aromatase activity in the hindbrain is higher in both females and type II males than in type I males.

These differences in aromatase expression correspond to differences in vocal behavior and its response to exogenous steroids. In type I males, intramuscular injection of the non-aromatizable androgen, 11-ketotestosterone (11KT) rapidly increased fictive call rate in type I males, while in females and type II males, estradiol (E2) and testosterone (T) rapidly facilitated the production of sex and morph specific calls. When aromatase was inhibited pharmacologically with fadrozole (FAD), only females showed disruptions in call duration (Remage-Healey and Bass, 2004, 2007; Shaw, 2018). This suggests that aromatase plays a key role in regulating sex and morph specific behavior by shifting local steroid concentrations toward estrogenic pathways or away from androgenic pathways in females. All three morphotypes have elevated circulating testosterone levels in the breeding season, but type II males and females have the highest levels and do not exhibit humming calls. (Only type I males show elevated circulating levels of 11KT). Aromatase is found abundantly in type II males and females may increase local estrogen concentration in the VMN, and thereby inhibit vocal activity (Forlano et al., 2005; Fergus and Bass, 2013; reviewed in Shaw, 2018). Alternatively, high aromatase levels in females may decrease VMN testosterone levels that appear crucial for supporting the production of humming vocalizations (Schlinger et al., 1999; Forlano and Bass, 2005a,b; reviewed in Shaw, 2018).

Brain aromatase may influence perception as well as the production of vocal signals. During the breeding season, females enhance the sensitivity of their auditory system to match the dominant frequency in the male advertisement call (Sisneros, 2009; reviewed in Shaw, 2018). Experimental treatment with either estradiol or testosterone induced this same shift in the auditory system (Sisneros et al., 2004; Shaw, 2018). The auditory nerve ganglion, located adjacent to the sensory epithelium of the inner ear’s saccule, expresses high levels of aromatase (Forlano et al., 2005; reviewed in Shaw, 2018). These observations suggest that aromatase increases local levels of estradiol in the ganglion *via* conversion of circulating testosterone, which then diffuses to the saccule to induce a cascade of events that shift the tuning of saccular hair cells (also see Rohmann et al., 2013).

#### Electric Fish

In contrast to vocal fish, electric fish show more ambiguous evidence for a direct effect of aromatization on the brain nuclei controlling signal production or reception. In a transcriptomic of *Apteronotus leptorhynchus*, Smith et al. (2018) found abundant aromatase transcripts in the hindbrain pacemaker nucleus (PN) that drives the continuous EOD and sets its discharge frequency. However, Shaw and Krahe (2018), using *in situ* hybridization, found no aromatase mRNA in the PN. These contrasting findings may result from different methods or from different gonadal states of the subjects. The midbrain prepacemaker nucleus (PPn-C) that regulates chirping behavior lacks aromatase mRNA, but the forebrain nuclei (e.g., the ventral subdivision of the ventral telencephalon, the POA and lateral hypothalamus) that influence electric signaling express abundant aromatase, suggesting that local estrogen production could indirectly affect communication by acting on higher order inputs to the electrocommunication circuitry (Shaw and Krahe, 2018).

Although it is not clear whether aromatase plays a prominent role in the regulation of electrocommunication, it clearly participates in the regulation of the unusual non-breeding aggressive behavior of the electric fish *Gymnotus omarorum*. In the past, aggression has been typically studied in the context of male competition for resources and mates during the breeding season, when elevated androgens produced by the gonads act on neural circuits in the brain (reviewed in Cunningham et al., 2012; Fuxjager et al., 2017). However, such reproduction-related male aggression is only one form of aggression. In some species, including *G. omarorum*, males display aggression during the non-breeding season as well as the breeding season, and females display aggression as well as males (Batista et al., 2012; Silva et al., 2020). In both of these unusual forms of aggression, circulating androgens are at low levels (Quintana et al., 2016). This non-breeding season male aggression and female aggression raise the question of how this behavior is regulated through mechanisms other than gonadal androgens. Several recent sets of studies suggest that such aggression is likely regulated through aromatization of extra-gonadal androgen into estrogen (Jalabert et al., 2015).

While *G. omarorum* shows aggression in both the breeding and non-breeding season, the underlying mechanisms appear to vary seasonally. During the non-breeding season, male aggression is unaffected by castration, and dominant and subordinate males do not differ in plasma levels of 11-KT. Thus, non-breeding aggression is independent of androgens or any other gonadal signal. However, treatment with an aromatase inhibitor, FAD, rapidly (within 30 min) decreases aggression, indicating that production of estrogens in the brain act through quick non-genomic mechanisms to regulate non-breeding aggression (Jalabert et al., 2015). In contrast to this non-breeding aggression, aggression during the breeding season most likely depends on the more typical hormonal regulation: high circulating androgen levels originating from the gonads increase aggression with a time course of hours to days. Thus, while the aggressive behavior of males is similarly high all year, the underlying hormonal control mechanisms change seasonally (Quintana et al., 2016).

Female agonistic behavior during the non-breeding season depends on aromatase in a manner similar to that in males. FAD treatment to females rapidly inhibits overall female aggression. Notably, treatment with an androgen receptor antagonist does not affect aggressive levels, at least over the timescale of minutes (Zubizarreta et al., 2020). These studies indicate that estrogen originating in the brain regulates aggression in females as well as males. None of these aromatase-dependent changes in overt aggression are accompanied by changes in electric signaling (Zubizarreta et al., 2020).

#### Comparison

Studies of aromatase have expanded our notions of how steroids regulate social behavior in these two teleost groups. In vocal fish, such studies have helped explain how steroids can have rapid effects on vocal behavior by the local production and rapid action of estrogens, especially in females. In electric fish, these studies have helped explain the regulation of female aggression and non-breeding male aggression. However, while there is abundant neuroanatomical and behavioral evidence for a direct action of aromatase on the vocal nuclei of vocal fish, the evidence for a direct action in the electrocommunication system is still equivocal.

### Social Regulation of Steroids and Communication Behavior

Before 2005, several studies in these two systems focused on how seasonal changes in the *physical* environment stimulate steroid production, which then had long-term actions on the nervous system to cause seasonal changes in reproductive behavior and signaling. More recently, two sets of studies have demonstrated that specific features of the *social* environment can induce steroid secretion and consequent changes in social behavior. One commonality in these studies is that they demonstrate that in both electric and acoustic modalities, exposure to communication signals alone is sufficient to induce steroid-dependent changes in behavior.

#### Vocal Fish

As male toadfish gradually populate nesting sites during the breeding season, the calling of one male can induce calling in neighboring males. Field experiments showed that non-calling males can be induced to call within 48 h by exposing them to a nearby calling male (Remage-Healey and Bass, 2005). Such social exposure elevated plasma 11KT levels without affecting plasma cortisol. Subsequent fieldwork demonstrated that audio playbacks of male calls were sufficient to elicit this behavioral and hormonal response. This response was only elicited by acoustic stimuli that replicated the naturally occurring advertisement call (“boatwhistle”) and not by less realistic acoustic stimuli. Further studies showed that experimentally increasing 11KT levels in non-calling males by feeding them food pellets embedded with 11KT increased call rate and duration within 20 min (Remage-Healey and Bass, 2006). Underwater audio playbacks induced an increase in both call rate and duration, implying a separate effect of auditory stimulation on call duration (Remage-Healey and Bass, 2005). The rapid effect of this treatment along with companion neurophysiological studies indicated that androgens exert their action through a non-genomic mechanism (Remage-Healey and Bass, 2006). Together these studies support a model of social regulation of communication in which acoustic features of the natural call cause an increase in androgen secretion which then potentiates calling by acting rapidly on nuclei of the vocal network.

#### Electric Fish

In the wild, male electric fish, *Apteronotus leptorhynchus*, emit chirps when intruder males enter their territory and compete for mates (Henninger et al., 2018). In the laboratory, long-term exposure of a male to a nearby conspecific male potentiated chirping over a time course of 4 days (Dunlap et al., 2002). Under these conditions, a male’s overall chirp rate decreased over time, but when presented with a standardized synthetic electric signal that mimics a conspecific male, the focal male chirped at greater rates, indicating that the underlying neural circuitry becomes sensitized to stimuli. Such long-term social interactions simultaneously increased plasma cortisol without affecting androgens. Experimental treatments that increased cortisol in isolated males potentiated chirping while pharmacologically blocking cortisol receptors in socially exposed males decreased chirping (Dunlap et al., 2011). These hormonal manipulations indicate that cortisol causally contributes to socially induced changes in chirping behavior (Dunlap et al., 2013).

**FIGURE 4:**
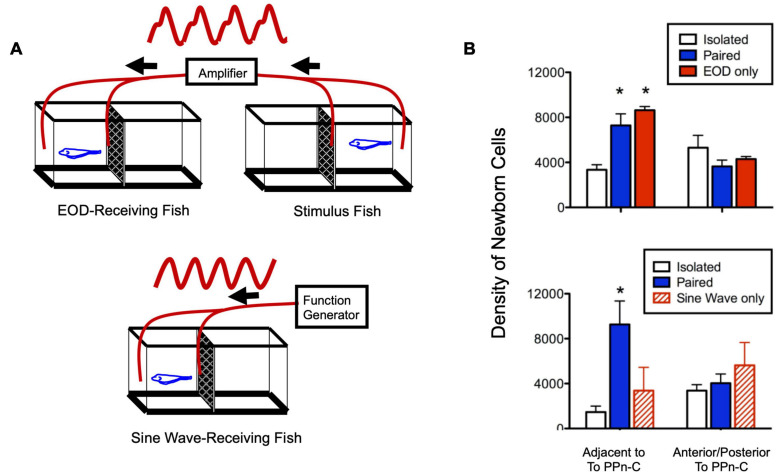
Social electric signals stimulate neurogenesis in the electrocommunication circuitry. **(A)** Experimental set up in which fish received a real, one-way social electric signal from a conspecific (top) or an artificial sine-wave stimulus (bottom). **(B)** Fish exposed to direct paired social interaction or EOD stimulus alone have equivalently higher levels of neurogenesis compared to isolated fish in a neurogenic zone adjacent to the brain region controlling chirping (prepacemaker nucleus, PPn-C), but not in a neighboring control region (top). Fish receiving an artificial sine-wave stimulus show levels of neurogenesis equivalent to isolated fish (bottom). Asterisks indicate significantly different than isolated group. Adapted and modified from Dunlap et al. (2008).

In addition to their effect on chirping behavior, social exposure and cortisol treatment increased the addition of newborn neurons in the PPn-C, the brain region that regulates chirping (Dunlap et al., 2006). While the precise mechanism by which this neurogenesis potentiated chirping is not known, the temporal and regional specificity of the effect strongly suggests that it contributes to socially induced, cortisol-dependent changes in chirping behavior (Dunlap et al., 2013). Experimental presentation of electrocommunication signals alone was sufficient to induce these changes in the neurogenesis and behavioral output of the PPn-C, but a simple electrical sine wave of the same frequency was ineffective (Figure 4; Dunlap et al., 2008). Thus, like in vocal fish, this behavioral change can be elicited with stimuli in a single modality, and only when these stimuli quantitatively mimic the natural communication signal.

#### Comparison

Studies in both vocal and electric fish have thus identified specific components of social signals that are effective in causing steroid-mediated changes in social behavior. However, these studies have focused on different behavioral contexts, timescales, steroids and neural mechanisms. In vocal fish reproductive signaling, social stimuli rapidly elevate androgen levels which activate the vocal-motor circuits within minutes through non-genomic pathways. In electric fish aggressive signaling, social stimuli elevate cortisol levels which promote new cell formation in the PPn-C to modify the output of the electrocommunication over the course of days.

## Contributions of Molecular Studies to the Neuroethology of Social Behavior

Since the 2005 review (Bass and Zakon, 2005), many new molecular techniques, particularly in transcriptomic analysis, have enabled neuroethologists to probe genetic mechanisms underlying social behavior. While work at this molecular level is still in its infancy in both systems, several studies have demonstrated new ways that the neural circuitry underlying vocal and electric communication are regulated over both physiological and evolutionary timescales.

### Vocal Fish

Recent studies using contemporary transcriptomic techniques in midshipman have examined how the neuropeptide galanin, acting in the preoptic area (POA), influences neuroendocrine characters related to alternative reproductive tactics (ARTs). Both the POA and anterior hypothalamus (AH) of teleosts include neuronal populations comparable to populations of hormone-synthesizing neurons in the POA of birds and mammals. This region is referred to as the POA-AH in Tripp et al. (2018); but here it is referred to as the POA (for further discussion, see Tripp et al., 2020). Goodson and Bass (2000a) and Kittelberger et al. (2006) had previously provided strong neurophysiological evidence that the POA is a key node regulating expression of ART-related vocal behaviors in midshipman fish.

Propelled by advances in next generation sequencing technologies (RNA-seq), a recent transcriptomic study in midshipman fish revealed candidate genes related to hormone action in the POA that were specific to male morph (type I vs. type II) and behavior (nest-holding vs. cuckolding) (Tripp et al., 2018). Four genes – galanin, urocortin, corticotropin releasing hormone (CRH), and oxytocin receptor – showed highest expression levels in courting type I males, which provide parental care, compared to both type I and type II cuckolders, which do not provide parental care. Two other genes, thyrotropin and growth hormone, showed the highest expression levels in cuckolding type I and II males compared to courting type I males.

**FIGURE 5:**
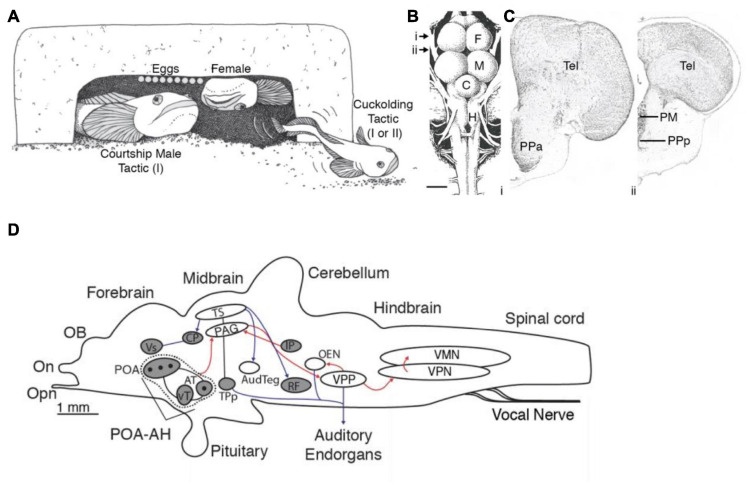
Alternative male reproductive morphs, preoptic area and galanin expression in the vocal circuitry of plainfin midshipman, *Porichthys notatus.* **(A)** Male midshipman exhibit alternative patterns of reproductive tactics. Type I males guard nests under rocky shelters, from where they broadcast a long duration (up to 2 h), multi-harmonic advertisement call known as a hum to attract females for spawning. Type II males do not exhibit these behaviors, but instead satellite (as shown here) or sneak (within the nest) spawn trying to steal egg fertilizations from resident type I male. Type I males that are small in body size also sneak or satellite spawn when they are unable to have their own nest. See text for more details. **(B)** Dorsal view of midshipman brain. Arrows indicate level of sections shown to the right. Scale bar represents 1 mm. **(C)** Transverse sections through midshipman brain at rostral (i) and caudal (ii) levels of the preoptic area (see panel **B**). **(D)** Distribution of immunoreactive galanin expression differs between male and female midshipman. Figure shows sagittal view with major nuclei in the auditory and vocal systems. Black dots indicate location of Gal-ir somata. Shading indicates brain regions having Gal-ir fibers in females and both male morphs, whereas unshaded regions contain Gal-ir fibers in both male morphs that are greatly reduced or absent in females. Red and blue lines indicate connections within the vocal and auditory systems, respectively. Arrowheads show direction of connections. Lines without arrowheads indicate reciprocal connections. C, cerebellum; F, forebrain; H, hindbrain; M, midbrain; PM, magnocellular preoptic area; PPa, anterior parvocellular preoptic area; PPp, posterior parvocellular preoptic area; Tel, telencephalon. Adapted from Tripp and Bass (2020) and Tripp et al. (2020).

The well-described influence of galanin on social behavior (including parental and sexual behavior) in rodents (Bloch et al., 1996; Park and Baum, 1999; Moffitt et al., 2018) inspired Tripp and Bass (2020) to follow up the transcriptomic analysis with two subsequent studies. The first study mapped the distribution of galanin throughout the brain using a midshipman-specific galanin antibody and revealed a sex difference in the number of galanin-containing somata in the POA and the density of galanin-labeled fibers, especially in the midbrain and the hindbrain (both values were greater in both male morphs than in females) (Figure 5; Tripp and Bass, 2020). The results supported the earlier transcriptome study, showing that the POA has the largest population of galanin-containing somata in the brain (see references in Tripp and Bass, 2020 for other teleosts).

In the second study, Tripp et al. (2020) used the galanin antibody together with a marker for neural activity, phosphorylated S6 protein (pS6; see Knight et al., 2012), to determine whether morphotype or vocal behavior correlated with activation of galanin-containing neurons. They found a far greater proportion of active galanin-containing POA neurons in courting type I males than in females and cuckolding type I and type II males. Moreover, this greater fraction of active galanin neurons was found only when courting type I males were in the nest with gravid females and not when they were either guarding previously fertilized eggs or defending the nest against type I or II cuckolders (Tripp et al., 2020). Thus, the activity of these galanin-containing neurons is specific to both morphotype and behavior. The results are consistent with earlier studies using microarray and RNA-seq analyses of whole brain samples in other teleost species with (bluegill sunfish *Lepomis macrochirus*; Partridge et al., 2016) and without (African cichlids, *Astatotilapia burtoni*, formerly *Haplochromis burtoni*; Renn et al., 2008) ARTs and suggested a role for galanin in the regulation of divergent patterns of social behavior among males. The lack of increased activation of galanin-containing neurons during egg care by type I males is consistent with the results in a study of dendrobatid poison dart frogs showing a similar lack of response in species with uniparental (male or female) care, whereas one species with biparental care shows increased galanin-POA neuron activation during parental care (Fischer et al., 2019). In aggregate, these studies suggest that POA-galanin neurons (1) have a conserved role in reproductive-related behaviors in lineages as divergent as fish, amphibians and mammals, and (2) are one of the neural substrates contributing to the evolution of ARTs among teleosts and perhaps other vertebrates (see Tripp et al., 2020 for further discussion).

These investigations point to exciting new research directions to pursue in the future, for example those investigating interactions between galanin and other hormone signaling systems recognized in the initial transcriptome study (Tripp et al., 2018). How might those signaling mechanisms change the intrinsic and network properties of neuronal networks driving social behaviors, such as reproductive-related vocalization in midshipman fish?

### Electric Fish

In electric fish, molecular work has focused mainly on the circuitry that generates the EOD (Pn, spinal electro-motoneurons [EMNs], and EO), which are evolutionarily novel structures. The earliest studies, begun before the widespread use of transcriptomics, took a candidate-gene approach focusing on species differences in expression and sequence of a muscle-expressing, voltage-gated sodium channel (*scn4a*) gene. This gene duplicated in an ancestral teleost, and eventually one paralog (scn4aa) shifted its expression from muscle to the evolutionary novel, muscle-derived EO in both mormyroids and gymnotiformes (Zakon et al., 2006; Arnegard et al., 2010; Paul et al., 2016). There, it evolved rapidly and likely contributed to the underlying species differences in EOD.

**FIGURE 6:**
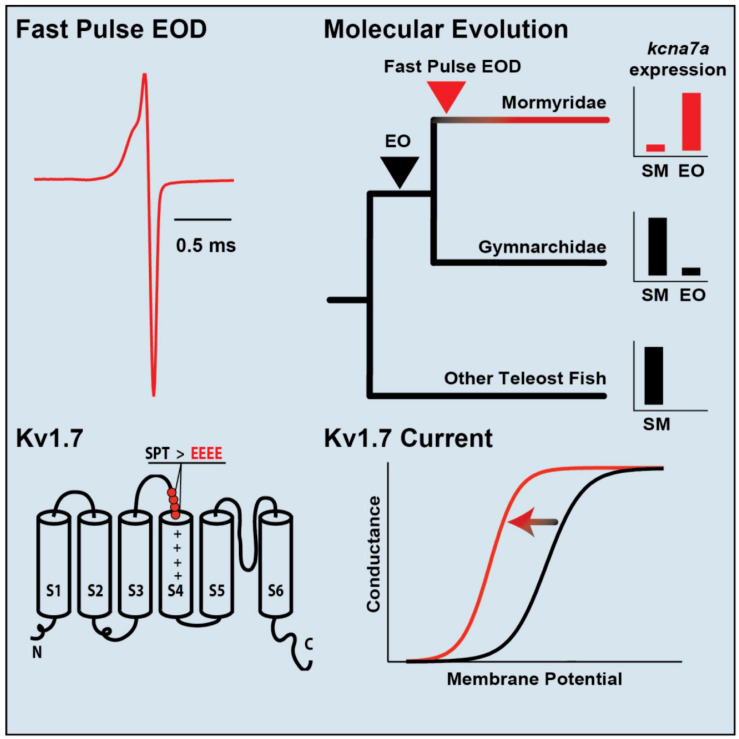
Specialization of a voltage-gated potassium channel for tuning species-specific EOD duration. **(Top, left)** The mormyrid *Brienomyrus brachyistius* produces a brief EOD pulse about half a millisecond in duration. **(Top, right)** the potassium channel gene, kcna7a (which makes a potassium channel called Kv1.7), is expressed in skeletal muscle (SM) in most teleosts (black), but it has shifted its expression to the electric organ (EO) in mormyrids (red). **(Bottom, right)** Sensitivity of Kv1.7 channel to voltage shown by a sigmoidal curve of membrane potential vs. conductance. The sensitivity of Kv1.7 in *B. brachyistius* (red) is shifted to the left (i.e., is activated at a more hyperpolarized voltage) than in other teleosts (black). **(**Bottom**, left)** The cause of the leftward shift in the membrane potential-conductance curve is due to the evolution of four negatively charged glutamates (EEEE) that replaced three neutral amino acids (SPT, serine, proline, threonine) in an ancestral mormyrid. This has occurred above the voltage-sensing part of the potassium channel (S4) and indicated by the plus signs. Adapted and modified from Swapna et al. (2018).

Subsequent transcriptomic studies assess differences in gene expression between muscle and EO more broadly (Gallant et al., 2014, 2017; Nagel et al., 2017). One recent study utilized the rapidly radiating mormyrid genus *Paramormyrops* to identify a gene for structural elements of the EO that vary across species and might be the basis for species differences in EOD waveform (Losilla et al., 2020). Transcriptomes of mormyrid EOs also revealed a gene for a voltage-gated potassium channel (*kcna7a*) that is expressed at high levels (Figure 6; Swapna et al., 2018). Like the sodium channel gene *scn4aa*, this gene is expressed in muscle of other fish but shifted its expression into the EO in the ancestor of mormyrids and underwent a burst of rapid evolution. This channel evolved a novel region that shortens action potential duration, thereby shaping the extremely brief EODs characteristic of many mormyrid species.

Most work on the evolution of electric signaling in the gymnotiformes comes from the family Apteronotidae. Apteronotids are interesting for a few reasons. First, they have a neurogenic electric organ, that is formed by the axons of EMNs. Second, as mentioned above, their EMNs are spontaneously active and synchronized by descending inputs from the Pn. Third, they have strong sex differences in EOD frequency and the direction of sexual dimorphism differs across species. In a transcriptomic analysis of the PN, Smith et al. (2018) identified a number of genes that are differentially expressed between two species of apteronotids with species differences in EOD range and the direction of sexual dimorphism. These include genes for steroid receptors and enzymes in steroidogenic pathways, as well as various ion channels that likely control the continuous firing frequency of PN neurons. Thompson et al. (2018), identified a novel voltage-gated Na+ channel (*scn4ab1*) expressed in the EMNs that resulted from a gene duplication within apteronotids. This channel has amino acid substitutions that prevent it from closing completely. Continuous Na+ influx through this leaky channel leads to spontaneous firing of the EMNs.

Just recently, researchers have begun using molecular analysis to examine how evolutionarily novel regions of the brain originated and how these new sensory and motor regions interface with the existing brain regions controlling social behavior (e.g., hypothalamic nuclei). As a start to this endeavor, Eastman et al. (2020) examined gene expression in the hypothalamus of a gymnotiform pulse-type species, *Gymnotus omarorum*. As mentioned above, this species is highly aggressive, even in the non-breeding season, and shows strong dominance-submissive relationships when paired in the laboratory. In this study, gene expression in the POA was assessed and a number of genes (such as somatostatin) and genes associated with sex steroid synthesis (aromatase) or metabolic processing (e.g., Cyp450) were differentially expressed between dominant and submissive animals.

The diversity of electric fish life histories and communication within each lineage and, especially the fact that numerous lineages evolved electroreception and electrogeneration, provides a richness for future mining using transcriptomic approaches, and the number of additional molecular techniques available for these groups is rapidly increasing (Pitchers et al., 2016).

## Vocal and Electric: The Neuroethology of Dual Communication Systems in Catfish

**FIGURE 7:**
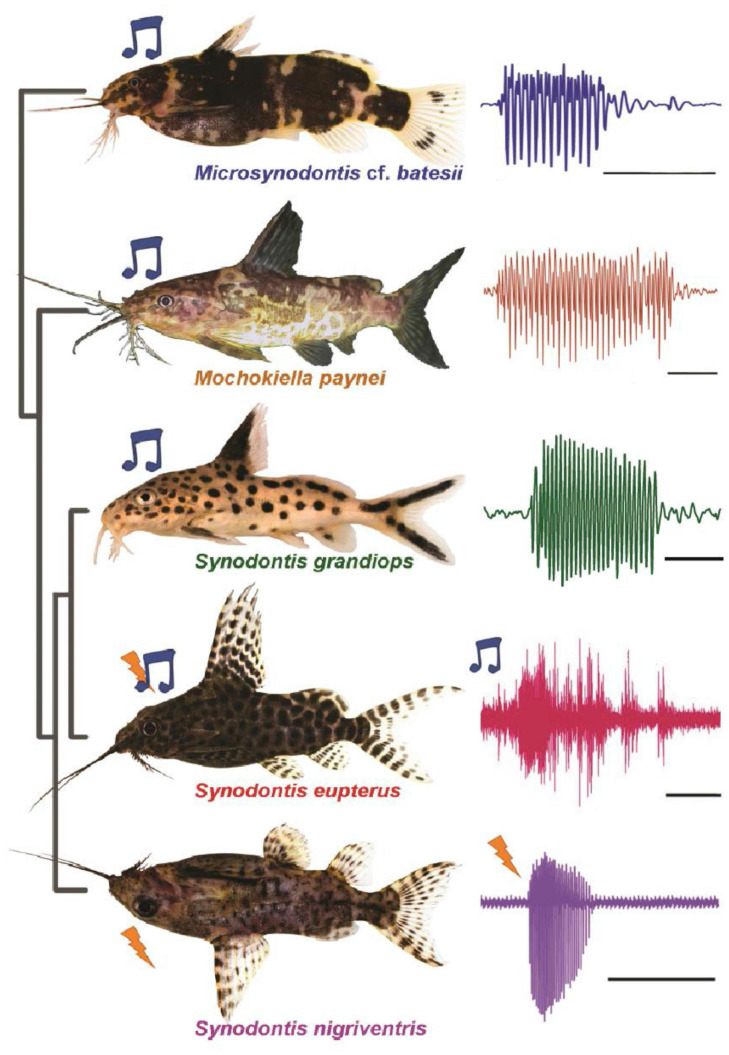
Mochokid catfish produce either vocal and/or weakly electric discharges. **(Left)** Photographs of five different mochokid species (blue note symbol denotes species that produce sound signal; lightning symbol denotes species that produce electric signal). Species studied so far are either sonic (e.g., top three), generate both types of signals (e.g., *S. eupterus*; only sonic signal shown here) or generate only electric discharges (e.g., *S. nigriventris*). **(Right)** Examples of waveforms of signals produced by the elastic spring apparatus. Scale bar is 50 ms for top four species shown and 500 ms for *S. nigriventris*. Modified and adapted from Kéver et al. (2020) and Kéver et al. (2021).

Mochokid catfishes offer a unique opportunity to reveal general principles underlying the organization of different communication systems because this speciose taxon includes species that produce either vocal signals or weakly electric discharges (ED) using the “same” neural circuitry and muscle (Figure 7; e.g., Hagedorn et al., 1990; Baron et al., 1994). In some especially intriguing species, a single individual can produce both vocal and electric signals from the same peripheral effector organ (Hagedorn et al., 1990). Some mormyrid fish are both weakly electric and vocal, but unlike mochokids, they use completely different organ systems to generate each type of signal (Bass, 1986; Crawford and Huang, 1999).

### Organization of Motor System in Catfish

Both vocal and electric signals are generated by the muscle associated with the elastic spring system (ESS), which evolved originally as a sonic swim bladder mechanism (Boyle et al., 2014). The ESS is composed of a neural circuit in the caudal hindbrain and the elastic spring apparatus (ESA) in the periphery. The ESA consists of the protractor muscle connecting a process of the fourth vertebra, the Müllerian ramus, and the swim bladder wall (Parmentier and Diogo, 2006). Contraction of the protractor muscle at pulse repetition rates of ∼100 Hz vibrates the swim bladder to generate sounds in a manner similar to the muscle surrounding the swim bladder of toadfishes.

Electrogenic catfish have smaller Müllerian rami compared to their vocal relatives (Kéver et al., 2021) and marked differences in the ultrastructure of the protractor muscle. In sound-producing species, this muscle has many myofibrils organized into highly ordered sarcomeres like other skeletal muscles, while in ED producing species, the muscle is largely missing this pattern of contractile elements (Boyle et al., 2014), with myocytes that resemble the independently evolved myogenic electrocytes of gymnotiform and mormyrids. When the protractor muscle of an ED producing species is treated with an acetylcholine antagonist, the amplitude of the ED is greatly diminished, indicating that the ED is indeed produced by activation of these modified muscle cells. Thus, the same muscle appears to function for sound production or electric discharge (Boyle et al., 2014).

The protractor muscle is innervated by the hindbrain motor nucleus, which receives input from several premotor neural populations (Hagedorn et al., 1990; Ladich and Bass, 1996; Kéver et al., 2020, 2021). The overall organization of this circuit is similar to the comparable circuit in toadfishes (see section “Neural Circuitry Generating Vocal and Electric Signals” above): a medially fused nucleus with large motoneurons and surrounding premotoneurons.

Many species of mochokid catfish produce either vocal or electric signals. However, some, such as *Synodontis eupterus*, can produce both vocal and electric signals in different phases of social behavior. In *S. eupterus*, the ESS phenotype (e.g., the density of myofibrils in the protractor muscle and the length of the Mullerian ramus) is intermediate between vocal-only or electric-only species (Hagedorn et al., 1990; Boyle et al., 2014; Kéver et al., 2021). In addition, the neural circuit that controls the ESS, has a larger pool of motoneurons compared to the homologous circuit in closely related species that produce only sonic or only electric signals. Thus, while this dual signaling species has evolved an “intermediate” peripheral signaling organ, it has simultaneously evolved greater motor control by the brain.

At higher levels in the control circuit, little is known about the neurochemical identity of the transneuronally mapped premotoneurons (i.e., excitatory, inhibitory or modulatory) or how the intrinsic and network properties of neurons contribute to motor patterning of protractor muscle output, whether sonic or electric. Behavioral and EMG recordings from the protractor muscle, however, indicate precise bilateral synchronous contractions, with high repetition rates suggestive of superfast muscles in the vocal species (Rome et al., 1996; Rome, 2006). A study investigating differences between the intrinsic properties of motoneurons of a vocal and ED fish showed that indeed motoneurons are adapted to such precise firing (Kéver et al., 2020). Electrophysiological studies like those carried out in toadfishes are needed to better understand the function of the individual network components.

### Evolutionary Patterns in Vocal and Electric Communication in Catfish

The available phylogenetic evidence suggests that vocal signaling is the ancestral condition among mochokid catfishes (Kéver et al., 2021). However, many of the investigated species in the genus *Synodontis* appear to have transitioned partly or entirely to electric signaling (see above). The ability to communicate with multiple channels might be selectively advantageous to both sender and receiver. But what selection pressures favored a full transition from vocal to electric or the ability to generate both signaling modalities? While all fishes can apparently hear sounds, only some (including catfishes) have the capability to detect weakly electric fields (Andrianov and Ilyinsky, 1973; Peters and van Wijland, 1974; Knudsen, 1976a,b; Bullock and Heiligenberg, 1986; Peters and van Ieperen, 1989). Thus, communication in the electric modality would offer a more “hidden” form of communication and limit detection by non-electroreceptive predators. Environmental factors could further favor such transitions. As suggested by Kéver et al. (2020), clear water environments could favor the more cryptic ED system since the combination of acoustic and visual signals in clear water would make them especially conspicuous to predators. In addition, the two modalities differ considerably in their effective communication distance: EDs are short range signals while acoustic signals are far ranging (Heiligenberg, 1977; Brenowitz, 1986; Rogers and Cox, 1988; Bass and Clark, 2003). EDs could thus be favored for close range communication (<1 m), while vocal signals could be favored for longer-distance communication (>1 m). While many questions about mochokid catfish remain unexplored, they might offer insights to fundamental issues in comparative neuroethology, such as the developmental and evolutionary origins of novel communication channels among vertebrates.

## Future Directions

Through over 50 years of intensive research in these two systems, we are now well aware of the many commonalities and differences in the regulation of circuits underlying vocal and electric communication in teleost fish. Still, the usefulness of this comparison would be advanced by future research in several specific areas. For example, more thorough mapping of neuromodulator receptors in electric fish, particularly receptors for 5HT and melatonin, would enable us to compare the neuromodulatory regulation of electromotor and electrosensory systems with the better mapped receptor systems of vocal fish. On the other hand, additional research on the physiological actions of 5HT on vocal circuitry and behavior and the behavioral actions of 5HT and AVT on aggressive behavior would allow for better comparison with similar published studies in electric fish. Currently, there is no information on the role of adult neurogenesis in the regulation of social behavior in vocal fish and little information on the role of galanin in the social behavior in electric fish; future research in both these areas will allow for interesting comparisons. Finally, although the role of light and melatonin in regulating daily cycles of social behavior have been examined in both species, very little is known in either system about their role in seasonal cycles of behavior.

Mochokid catfish with both vocal and electrogenic systems raise particularly interesting questions about the regulation of the neural circuitry underlying social communication. Do the same modulatory systems that regulate the more ancestral vocal system also regulate the more derived electrogenic system? Do the modulators act to control the same temporal patterning in both systems? In species that produce both vocal and electric signals, are neuromodulators involved in switching between these dual modalities? Comparative neuroanatomical, physiological and behavioral studies among mochokid catfishes offer many opportunities to investigate these questions.

While research on these important gaps in our understanding of vocal and electric communication in adult fish should be pursued, we also believe there is great promise in examining the ontogeny of neural circuits in these systems as well. Further application of transcriptomic methods, including single cell RNA sequencing, would allow us to characterize how certain cell types within a circuit (for example, pacemaker vs. vocal/electro- motoneurons) differ in gene expression. These techniques could similarly describe how the novel, highly specialized cells involved in vocal and electric communication diverge from ontogenetically and phylogenetically homologous tissue types (e.g., the transition from skeletal muscle to sonic muscles or electrocytes). Parallel developmental studies could trace when and how these differences arise during ontogeny. Recent methods in spatial transcriptomics (Waylen et al., 2020) could resolve gene expression differences in closely apposed cell types within the developing neural circuits. Finally, targeted genetic manipulations [e.g., using CRISPR (Constantinou et al., 2019) for loss-of-function studies or transgenics for gain-of-function studies] could then demonstrate which genetic differences contribute causally to the divergence in neuronal phenotype within a neural circuit or the emergence of novel cell types during evolution.

Catfish with dual-modality signaling systems offer a particularly interesting model for addressing how vocal and electric communication systems are constructed in other teleosts. Are similarities in the neural circuits that generate these different signals in the same individual attributable to a shared tissue origin or common developmental processes? How do differences in their vocal and electrogenic circuits emerge ontogenetically? In the broadest sense, these and other future investigations of vocal and electric fish offer great promise for those seeking to uncover mechanisms underlying the evolution and development of vertebrate social behaviors.

## Author Contributions

All authors contributed to the writing and editing of the manuscript.

## Conflict of Interest

The authors declare that the research was conducted in the absence of any commercial or financial relationships that could be construed as a potential conflict of interest. The handling editor declared a past co-authorship with one of the authors HZ.

## Publisher’s Note

All claims expressed in this article are solely those of the authors and do not necessarily represent those of their affiliated organizations, or those of the publisher, the editors and the reviewers. Any product that may be evaluated in this article, or claim that may be made by its manufacturer, is not guaranteed or endorsed by the publisher.
